# Development and validation of PEYRO-Q: a novel multidimensional patient-reported outcome measure for Peyronie’s disease

**DOI:** 10.1093/sexmed/qfag034

**Published:** 2026-05-18

**Authors:** Andrea Cocci, Mattia Lo Re, Marta Pezzoli, Javier Romero-Otero, Juan Ignacio Martinez Salamanca, Nicolas Mourel-Journel, Paul Neuville, Antoine Faix, Sam Ward, Koenraad Van Renterghem, Georgios Hatzichristodoulou, Daniar Osmonov, David Ralph, Anna Cadenar, Marco Falcone, Giorgio Ivan Russo, Carlo Bettocchi, Giovanni E Cacciamani, Nicola Mondaini, Marta Skrodzka, Edoardo Pozzi, Andrea Salonia

**Affiliations:** Department of Experimental and Clinical Medicine, University of Florence, 50100 Florence, Italy; Department of Experimental and Clinical Medicine, University of Florence, 50100 Florence, Italy; Unit Urology and Andrology, University of Florence, Careggi Hospital, 50100 Florence, Italy; Department of Experimental and Clinical Medicine, University of Florence, 50100 Florence, Italy; Unit Urology and Andrology, University of Florence, Careggi Hospital, 50100 Florence, Italy; Department of Urology, Hospital Universitario HM Sanchinarro, Instituto Investigación Sanitaria HM Hospitales and ROC Clinic, 28010 Madrid, Spain; Urology Department, Hospital Universitario Puerta de Hierro Majadahonda and Lyx Institute of Urology, Universidad Francisco de Vitoria, Madrid 28223, Spain; Urology Department, Hôpital Lyon Sud, Hospices Civils de Lyon, 69495 Lyon, France; Urology Department, Hôpital Lyon Sud, Hospices Civils de Lyon, 69495 Lyon, France; Department of Urology, Department of Andrology, Clinique du Millénaire, 34000 Montpellier, France; Department of Urology, Cliniques Saint-Jean, 1000 Bruxelles, Belgium; Department of Urology, Jessa Hospital, 3500 Hasselt, Belgium; Department of Urology, Martha-Maria Hospital Nuremberg, Nuremberg, Germany; Department of Urology, University Hospital Schleswig Holstein, Kiel 24105, Germany; Department of Urology, University College London Hospitals, Great Britain, London NW1 2PB, United Kingdom; Department of Experimental and Clinical Medicine, University of Florence, 50100 Florence, Italy; Unit Urology and Andrology, University of Florence, Careggi Hospital, 50100 Florence, Italy; Urology Clinic - A.O.U. "Città della Salute e della Scienza"- Molinette Hospital, University of Turin, 10126 Turin, Italy; Urology Section, Department of Surgery, University of Catania, 95131 Catania, Italy; Biruni University, School of Medicine, Urology Department, Istanbul, Turkey; Andrology and Male Genitalia Reconstructive Surgery Unit, University of Foggia, 71122 Foggia, Italy; USC Institute of Urology, Catherine & Joseph Aresty Department of Urology, Keck School of Medecine University of Southern California (USC), Los Angeles, CA, United States; Department of Urology, University Magna Graecia, Catanzaro 88100, Italy; Department of Urology, St. Georges University Hospital, London SW17 0QT, United Kingdom; Vita-Salute San Raffaele University, Milan 20132, Italy; Division of Experimental Oncology/Unit of Urology, Urological Research Institute, IRCCS Ospedale San Raffaele, Milan 20132, Italy; Department of Urology, St. Georges University Hospital, London SW17 0QT, United Kingdom; Vita-Salute San Raffaele University, Milan 20132, Italy

**Keywords:** Peyronie’s disease, penile curvature, validated questionnaire

## Abstract

**Background:**

Peyronie’s disease (PD) is a condition with significant physical, sexual, and psychological impact that is not fully captured by existing Patient-Reported Outcome (PRO) instruments.

**Aim:**

To develop and preliminarily validate the PEYROnie’s disease Questionnaire (PEYRO-Q), a novel multidimensional PRO questionnaire designed to provide a comprehensive and inclusive assessment of PD.

**Methods:**

A prospective multicenter study was conducted between April and July 2025, enrolling 206 adult patients with PD. The PEYRO-Q, developed in accordance with COSMIN guidelines, includes 10 domains and 12 items assessing deformity severity and stability, pain, penile shortening, sexual function, erectile function, psychological distress, and sexual avoidance. Content validity was established through expert panel review. Internal consistency was evaluated using Cronbach’s alpha, while test–retest reliability was assessed over a 2-week interval. Exploratory Factor Analysis (EFA) with promax rotation was performed to investigate the underlying structure.

**Outcomes:**

Primary outcomes were internal consistency, test–retest reliability, and factor structure of the PEYRO-Q.

**Results:**

The study population had a median age of 62 years, with most patients reporting moderate penile curvature and long-standing disease stability. The PEYRO-Q demonstrated excellent internal consistency (Cronbach’s α = 0.90) and test–retest reliability (α = 0.89–1.00; Intraclass Correlation Coefficient, ICC up to 0.99). EFA supported a three-factor structure explaining 62.2% of total variance, corresponding to clinical/functional burden, disease perception and evolution, and psychological/behavioral impact. Moderate correlations between domains (eg, erectile aid use and psychological distress, r = 0.41) confirmed related but distinct constructs. The questionnaire allowed detailed and inclusive profiling of sexual function and psychological burden.

**Clinical Implications:**

The PEYRO-Q offers a practical, inclusive, and comprehensive tool for assessing PD burden in both clinical practice and research settings.

**Strengths & Limitations:**

Strengths include the multidimensional and inclusive design, strong psychometric performance, and multicenter prospective validation. Limitations include the predominantly Italian cohort, lack of longitudinal responsiveness data, absence of direct comparison with existing instruments, and limited clinical characterization such as plaque localization.

**Conclusion:**

The PEYRO-Q is a reliable and clinically relevant PRO instrument for PD, warranting further validation to confirm responsiveness and cross-cultural applicability.

## Introduction

Peyronie’s disease (PD) is an acquired connective tissue disorder characterized by the formation of fibrotic plaques within the tunica albuginea of the corpora cavernosa. This process may lead to penile morphometric changes such as curvature, shortening, retraction, hourglass deformities, or hinge effects, and is frequently associated with painful erections during the active phase or with varying degrees of erectile dysfunction (ED) in the chronic/stable stage.^1–3^ The global incidence of PD is estimated at ~25 per 100 000 men, with most of cases occurring between the ages of 45 and 60.^4^

The psychological and functional impact of PD is often underestimated. Studies have shown that men affected by PD frequently experience reduced self-esteem, sexual avoidance, relationship distress, and symptoms of anxiety or depression.^5^ Consequently, accurate evaluation of the multifaceted burden of the disease is essential to guide clinical decision-making and monitor therapeutic outcomes.

In recent years, several instruments have been developed to assess sexual function and quality of life in men with PD, with the Peyronie’s Disease Questionnaire (PDQ) being the most widely validated and used internationally.^6^ The PDQ was designed according to Food and Drug Administration (FDA) guidelines to capture penile pain, physical and psychological symptoms, and overall symptom bother.

However, despite its utility in clinical trials, the PDQ presents several limitations that hinder its widespread use in clinical practice and diverse populations. These include a rigid focus on heterosexual, vaginal intercourse; the lack of grading for curvature severity or disease phase (active vs. stable); and a structure that may be time-consuming or perceived as redundant by patients. Additionally, licensing constraints limit its application in independent research and non-commercial settings.

Considering these limitations, we introduce the PEYROnie’s disease Questionnaire (PEYRO-Q), a novel multidimensional patient-reported outcome (PRO) instrument specifically developed for individuals with PD. The PEYRO-Q is designed to deliver a comprehensive, inclusive, and easily interpretable assessment of the physical, sexual, and psychological impacts of PD. Unlike previous tools, it captures the full spectrum of deformity severity, accounts for disease phase, incorporates non-penetrative sexual activity, and accommodates patients of all sexual orientations.

This paper details the development and preliminary validation of the PEYRO-Q, including its content framework, internal consistency, and test–retest reliability, within a multicenter cohort of patients with PD.

## Materials and methods

From April 01, 2025 to July 31, 2025, a total of 206 patients were consecutively recruited from the outpatient clinic during the study period, and eligibility was confirmed according to predefined inclusion criteria (age ≥ 18 years, ability to provide informed consent, and absence of cognitive impairment interfering with questionnaire completion). After obtaining written informed consent, each participant was asked to complete a standardized questionnaire designed to evaluate clinical, functional, and patient-reported outcomes relevant to the study objectives.

The questionnaire was administered in a controlled environment, either in paper form or electronically, under the supervision of trained research personnel who ensured completeness of responses without influencing patient answers. Participants were instructed to answer independently and anonymously to minimize response bias. Of the total recruited patients, 206 provided fully completed and analyzable questionnaires, which were subsequently included in the statistical analysis.

### Description of the PEYRO-Q

The Italian PEYRO-Q consists of 10 domains and 12 questions (Table 1), each scored on an ordinal Likert-type scale (range 0-4, depending on the item), with higher scores indicating greater severity or impairment. The items were drafted in accordance with international recommendations for PRO development and underwent experts panel review for content validity.^7^ The development and validation of this tool followed best-practice guidance, including resources from the EQUATOR Network (COSMIN checklist for measurement instruments).^8^ The complete questionnaire is reported in original language as Supplementary Material 1.

**Table 1:** Conceptual domains of the PEYRO-Q questionnaire.

**Domain**	**Description**	**No. of items**	**Score range per item**	**Total domain score range**
Penile Curvature	Self-estimated severity of curvature during erection	1	0–4	0–4
Curvature Stability	Duration of stable curvature or deformity	1	0–4	0–4
Penile Pain	Intensity and temporal evolution of erection-related pain	2	0–4	0–8
Penile Shortening	Subjective perception of length reduction	1	0–4	0–4
Vaginal Intercourse Difficulty	Mechanical or positional challenges during vaginal sex	1	0–4	0–4
Anal Intercourse Difficulty	Mechanical or positional challenges during anal sex	1	0–4	0–4
Masturbation Difficulty	Limitations or discomfort during masturbation	1	0–4	0–4
Erectile Rigidity	Perceived firmness and adequacy of erection for penetration	1	0–4	0–4
Use of Erection Aids	Dependence on pharmacological or mechanical support for erections	1	0–4	0–4
Psychological Distress	Emotional, psychological, and relational burden of the deformity	1	0–4	0–4
Sexual Avoidance	Extent to which the deformity interferes with sexual activity	1	0–4	0–4

The domains were as follows:

1. Penile curvature severity – Self-estimated angular deviation during erection (0 = none, 4 = >90° or associated deformities such as hourglass or hinge effect).
2. Curvature stability – Duration of stable deformity (0 = still changing/worsening; 4 = stable >12 months).
3. Penile pain – Intensity of erection-related pain (0–10 scale, grouped into five ordinal levels), and its evolution over time.
4. Penile shortening – Subjective perception of length loss (0 = none; 4 = >3 cm).
5. Sexual function difficulties:

- Vaginal intercourse
- Anal intercourse
- Masturbation

1. Each assessed for mechanical difficulty or pain on a 0–4 scale (0 = no difficulty or no attempt; 4 = severe difficulty).
2. Erectile rigidity – Subjective ability to achieve and maintain penetration-quality erections (0 = full rigidity; 4 = completely flaccid).
3. Use of aids for erection – Reliance on pharmacological or mechanical devices (0 = none; 4 = no effect even with aids).
4. Psychological distress – Emotional and interpersonal burden (0 = none; 4 = severe, daily impact).
5. Sexual avoidance – Behavioral impact of PD on sexual activity (0 = no interference; 4 = total avoidance).
6. Pain evolution – Temporal change in pain symptoms over the preceding 6 months.

The PEYRO-Q is designed for application in both clinical practice and research, supporting baseline characterization, monitoring of disease progression, and assessment of therapeutic outcomes in observational studies and interventional trials.

The PEYRO-Q was developed following a structured, multi-step process. An initial pool of items was generated based on clinical expertise in andrology and a review of the existing literature on PD and patient-reported outcome measures. The items were designed to capture key domains of the disease, including anatomical changes, functional impairment, and psychological impact.

Content validity was assessed through an expert panel of urologists specialized in andrology, who evaluated each item for relevance, clarity, and comprehensiveness. Based on this process, items were refined to improve interpretability and clinical applicability.

Formal cognitive interviews with patients and structured item reduction procedures were not performed during this initial development phase. These aspects will be addressed in future studies aimed at further validating and refining the questionnaire in accordance with established methodological frameworks.

This paper reports the findings of a prospective, multicenter study. The experimental procedures were carried out in accordance with the Declaration of Helsinki.

The study was approved by local Institutions (CEAVC 29414), and written informed consent to participate in this study was obtained from each patient before enrolment.

### Questionnaire development and validation

Content validity was assessed by an expert panel of 22 urologists specialized in andrology, who reviewed the questionnaire for relevance, clarity, and comprehensiveness. All the panel involved have at least 5 years of proven experience in andrological surgery and minimally invasive therapy. The authors are affiliated with leading academic and clinical centers across Europe and North America, including institutions based in Italy, Spain, France, Belgium, Germany, the United Kingdom, Poland, and the United States, ensuring broad geographical representation and multidisciplinary expertise in the field of andrology and minimally invasive surgery. Consensus on items was reached using Content Validity Ratio and Content Validity Index. To evaluate internal consistency—ie, the extent to which all items measure the same underlying construct—we calculated Cronbach’s alpha coefficient. This statistic ranges from 0 to 1, with values above 0.70 generally considered acceptable for instruments based on Likert-type scales. According to the interpretation proposed by George and Mallery, values >0.90 are considered excellent, >0.80 good, >0.70 acceptable, >0.60 questionable, >0.50 poor, and < 0.50 unacceptable.^9^ To assess test–retest reliability, the questionnaire was administered twice, with a 2-week interval between administrations. Agreement between the two responses was evaluated using the weighted Cohen’s kappa coefficient (κ), which quantifies inter-rater reliability for ordinal variables. Kappa values were interpreted according to the Landis and Koch classification: <0.20 = poor, 0.21–0.40 = fair, 0.41–0.60 = moderate, 0.61–0.80 = good, and 0.81–1.00 = very good agreement.

Exploratory factor analysis (EFA) was performed on the baseline dataset using principal factor extraction with promax rotation.

All statistical analyses were conducted using Stata (StataCorp, College Station, TX, USA).

Test–retest reliability was assessed by administering the questionnaire to all participants on two separate occasions, with a 2-week interval between the first and second administration.

## Results

A total of 206 patients were included in the analysis. Median age of the population was 62 years (interquartile range [IQR]: 58-66). The majority of participants were aged between 55 and 64 years (39.3%), followed by those aged 65+ (25.2%) and 45–54 years (22.3%).

Among the 206 patients who completed the curvature-degree item, the majority (147 patients, 71.4%) reported a moderate penile curvature (grade 2). Mild curvature (<30°, grade 1) was reported by 22 patients (10.7%), whereas severe curvature (60–90°, grade 3) was reported by 22 patients (10.7%). A smaller subgroup, comprising 15 patients (7.3%), described a very severe curvature (>90° or associated with complex deformities such as hourglass or hinge effect, grade 4). Notably, none of the respondents reported an absence of penile curvature (grade 0).

The distribution of responses showed that 29 patients (14.1%) reported that their curvature was not yet stable and was still changing or worsening, while none of the patients indicated stability for ˂1 month. A total of 48 patients (23.3%) reported that the curvature had remained stable for 1–6 months, whereas 52 patients (25.2%) indicated stability lasting 6–12 months. The largest proportion, 77 patients (37.4%), reported stability for ˃12 months.

Patients were asked to report their ethnic or geographical background. The majority self-identified as Italian (84.5%), followed by other European (5.3%), North African (3.4%), and Sub-Saharan African (1.9%). Smaller proportions identified as Middle Eastern (0.5%), Asian (1.5%), or American (2.9%). No respondents selected “Oceanian,” “Other,” or “Prefer not to answer.”

Participants reported their highest level of completed education. The majority had completed upper secondary education (36.9%), followed by graduate degrees (18.0%) and undergraduate degrees (17.5%). A smaller proportion held a doctorate or postgraduate qualification (7.8%), while 5.3% reported no formal education. Only 0.97% chose not to disclose their educational level.

Sexual orientation was assessed via self-report using a five-category nominal item. The vast majority of participants identified as heterosexual (91.8%), while 5.3% identified as homosexual, and 1.0% as bisexual. A small proportion identified as “other” (1.5%) or preferred not to answer (0.5%).

Participants were asked to report their current employment status. The majority were employed full-time (63.1%), followed by retired individuals (17.5%) and part-time workers (6.3%). Smaller proportions reported being unemployed (5.8%), homemakers (1.9%), students (1.0%), or classified themselves under “other” (4.4%). No participants selected the option “prefer not to answer.”

Median total score was 19.26 (IQR: 13.5-24.75). Table 2 presents the results of the PEYROQ domain scores for the entire population. The Cronbach’s alpha of the questionnaire was 0.90. Test–retest reliability was assessed using Cronbach’s alpha for each PEYRO-Q item (Table 3). Cronbach’s alpha values ranged from 0.89 to 0.99, indicating temporal stability across all domains. Intercorrelation coefficients for the total score was 0.99 (IC 0.98-0.99).

**Table 2:** PEYRO-Q domain scores.

**PEYRO-Q item**	**Score, median [IQR]**
Penile curvature degree	2 [1–2]
Curvature stability	3 [2–4]
Erection-related penile pain (intensity)	0 [0–2]
Change in penile pain over the last 6 months	1 [0–2]
Penile shortening	1 [0–2]
Difficulty during vaginal intercourse	2 [1–3]
Difficulty during anal intercourse	0 [0-2]
Difficulty during masturbation	1 [0–4]
Erectile rigidity	1 [0–2]
Use of erection aids	0.5 [0–2]
Psychological distress related to penile deformity	2 [1–3]
Interference with sexual activity due to penile deformity	2 [1–3]

**Table 3:** Test–retest reliability of PEYROQ domains.

**Domain**	Cronbach’s alpha
Penile curvature degree	0.943
Curvature stability	0.975
Erection-related penile pain (intensity)	0.889
Change in penile pain over the last 6 months	0.987
Penile shortening	0.976
Difficulty during vaginal intercourse	0.978
Difficulty during anal intercourse	0.965
Difficulty during masturbation	0.944
Erectile rigidity	0.948
Use of erection aids	1.000
Psychological distress related to penile deformity	0.965
Interference with sexual activity due to penile deformity	0.975

Exploratory factor analysis (EFA) was performed on the baseline dataset using principal factor extraction with promax rotation. Bartlett’s test of sphericity was significant (χ^2^(66) = 281.76, *P* < .001), supporting the suitability of the data for factor analysis (Table 4).

**Table 4:** Exploratory factor analysis.

**Factor**	**Eigen value**	**Variance explained (%)**	**Cumulative variance (%)**
Factor 1	4.30	35.9	35.9
Factor 2	2.04	17.0	52.9
Factor 3	1.12	9.4	62.2

Based on eigenvalues, scree plot inspection, and clinical interpretability, a three-factor structure was retained. The first factor accounted for 35.9% of the variance, the second for 17.0%, and the third for 9.4%, with a cumulative explained variance of ~62.2%.

The rotated solution demonstrated a coherent and clinically interpretable structure. The first factor primarily reflected overall clinical and functional burden, including domains related to penile deformity, sexual function, and symptom severity. The second factor captured aspects related to disease perception and temporal evolution. The third factor was associated with psychological and behavioral dimensions, including distress and sexual avoidance.

To explore the relationship between the two domains, we performed a correlation analysis between the items assessing reliance on pharmacological or device support for erection (Domain P) and the psychological impact of penile deformity (Domain Q). Pearson’s correlation coefficient demonstrated a moderate positive association between the two domains (r = 0.41), indicating that greater reliance on supportive treatments was moderately associated with higher psychological burden. This finding was consistent when using Spearman’s rank correlation (ρ = 0.38). Importantly, both coefficients remained well below commonly accepted thresholds for redundancy (r > 0.75), supporting the interpretation that the two domains represent related but distinct constructs within the PEYRO-Q questionnaire.

## Discussion

The findings demonstrate that the PEYRO-Q is a reliable and consistent PRO measure, with strong internal consistency (Cronbach’s alpha = 0.90), exceeding the commonly accepted threshold of α > 0.70. Furthermore, the PEYROQ exhibited excellent test–retest reliability (intraclass correlation coefficient: 0.89–1.00), indicating its suitability for use in patients with PD.

As emphasized by Yazdi-Feyzabadi *et al.*, the development and validation of a questionnaire is a rigorous process governed by well-defined methodological standards; failure to adhere to these principles may result in an instrument that inadequately reflects the clinical characteristics of the disease.^7^

This is particularly relevant in PD, where evaluation of both psychological and sexual domains is of paramount importance.^5^ On one hand, sexual activity—when possible—is often described as more cumbersome and distressing^10^; on the other, altered penile appearance can undermine self-confidence and sexual self-efficacy, potentially exacerbating psychological distress.^11^

Given these considerations, a validated, accurate, and user-friendly instrument is essential to facilitate daily clinical practice and promote more consistent scientific reporting. In this context, the PDQ emerged to be a highly effective and reliable enough tool. Notwithstanding this, PDQ is not without limitations that have hindered its broader adoption in routine care and across diverse populations.

The PEYRO-Q was specifically designed to overcome these limitations, offering several key strengths that enhance both clinical and research applicability.

First, sexual inclusivity: unlike earlier tools, it includes domains assessing difficulties across various forms of sexual intercourse and masturbation, without presuming sexual orientation, thereby ensuring applicability to patients of all genders, orientations, and relationship statuses.

Second, comprehensive clinical profiling: it assesses the severity and stability of penile curvature, the presence and progression of penile pain, and distinguishes between active and stable disease phases, yielding a more nuanced clinical characterization.

Exploratory factor analysis supported the multidimensional nature of the PEYRO-Q, identifying a limited number of underlying latent factors that grouped clinically related items. Importantly, this structure reflects broader domains of disease burden rather than a need to reduce the number of questionnaire items. These findings reinforce the conceptual design of the instrument, which aims to capture the complex and interrelated anatomical, functional, and psychological dimensions of PD.

Finally, efficiency and clarity: the PEYRO-Q enables rapid yet comprehensive assessment, with clearly structured domains that both clinicians and patients easily interpret.

Despite these advantages, the study has several limitations.

First, although validation was conducted in a multicenter cohort, most participants were Italian (84.5%), which may limit the generalizability of the findings. Cross-cultural adaptation into other languages—such as the Spanish PDQ version by Borja García-Gómez *et al.*^12^ or the Italian adaptation by Traunero *et al.*^13^—should be undertaken in future research. Same concern must be reported regarded sexual orientation. Although the questionnaire was designed and written in a non-heteronormative manner, given that 91.8% of the patients were heterosexual, its validity cannot be formally extended to different sexual orientations. An additional validation study with a larger cohort will be necessary to confirm the validity of the questionnaire.

Second, the study employed a cross-sectional design with a 2-week retest interval. While this confirmed excellent temporal stability, the instrument’s responsiveness—its ability to detect clinically meaningful changes over time—remains to be assessed in longitudinal studies. Furthermore, the Minimum Clinically Important Difference (MCID) for the PEYRO-Q has not yet been established. The MCID represents the smallest change in score that patients perceive as meaningful and is essential for interpreting treatment effects in clinical trials and practice. Future studies should define the MCID through distribution-based approaches (eg, 0.5 standard deviation of baseline scores or the standard error of measurement) and anchor-based methods using external clinical criteria such as patient global impression of change, objective curvature improvement, or therapeutic interventions including collagenase *clostridium histolyticum* injections or surgical correction. Establishing the MCID will be a priority in the next validation phase of the PEYRO-Q.

Third, no direct comparison with other established instruments was performed; thus, concurrent validity was not evaluated and should be addressed in future work. A further limitation of the present study is the absence of data regarding plaque localization. The PEYRO-Q questionnaire was developed to capture patient-reported perceptions and subjective disease burden, and therefore did not include items aimed at assessing the anatomical characteristics of PD, such as plaque position or extent. As a result, the clinical phenotype of the cohort cannot be fully described, and the representativeness of the population with respect to different plaque locations cannot be determined. Future validation phases will incorporate objective clinical variables, including plaque localization, to enhance the comprehensiveness of the instrument.

Another limitation is the lack of exploratory factor analysis to formally investigate the dimensional structure of the instrument. Given the multidimensional clinical design of the PEYRO-Q, this will be addressed in future psychometric validation studies.

Finally, a relevant limitation of the present study is the absence of a direct comparison between the PEYRO-Q and existing validated instruments, such as the Peyronie’s Disease Questionnaire (PDQ). Although we sought permission to administer the PDQ, our request was not granted by the original authors, preventing its inclusion in the current analysis. As a result, external validation through convergent and divergent validity testing could not be performed at this stage. A dedicated second-phase study is planned to address this gap by evaluating the PEYRO-Q against widely used and accessible standardized questionnaires.

## Conclusions

The PEYRO-Q represents a novel, inclusive, and clinically relevant patient-reported outcome measure for PD. It demonstrates strong preliminary reliability and offers a comprehensive assessment of disease burden across multiple domains. Further validation studies are required to confirm its dimensional structure, responsiveness, and applicability across diverse populations.
